# The patterns of dynamic changes in blood toxicant levels, prognosis, and regression in a case of occupational chlorfenapyr exposure

**DOI:** 10.3389/ftox.2025.1570887

**Published:** 2025-06-06

**Authors:** Jianjian Liu, Zhaozhao Shan, Chen Wang, Xiangdong Jian, Baotian Kan, Yingli Ren

**Affiliations:** ^1^ Department of Poisoning and Occupational Diseases, Emergency Medicine, Qilu Hospital of Shandong University, Cheeloo College of Medicine, Shandong University, Jinan, Shandong, China; ^2^ Department of Occupational and Environmental Health, School of Public Health, Cheeloo College of Medicine, Shandong University, Jinan, Shandong, China; ^3^ School of Nursing and Rehabilitation, Cheeloo College of Medicine, Shandong University, Jinan, Shandong, China; ^4^ Department of Emergency Intensive Care Medicine Center, The Affiliated Hospital of Shandong University of Traditional Chinese Medicine, Jinan, Shandong, China

**Keywords:** chlorfenapyr, poisoning, event, toxicant detection, neurological imaging

## Abstract

**Objective:**

In July 2024, cases of chlorfenapyr poisoning occurred consecutively among workers in a chlorfenapyr production plant in Shandong Province. This study aimed to analyze the clinical characteristics of this event and discuss the significance of toxicant testing and imaging examinations in guiding clinical practice.

**Methods:**

We retrospectively analyzed the clinical data of four patients with occupational chlorfenapyr poisoning.

**Results:**

All four patients worked in the same factory before admission; three worked in the same workshop, while one worked in the product inspection department. Patients three and four had no obvious clinical manifestations, whereas patients one and two primarily presented with hyperhidrosis, fever, and neurological symptoms. Laboratory tests revealed abnormalities in blood counts, liver and kidney function indicators, and cardiac enzyme profiles in some patients. Magnetic resonance imaging revealed varying degrees of abnormal signal changes in the brain and spinal cord in Patients 1, 2, and 3. After comprehensive treatment with blood purification, organ protection, and symptomatic treatment, chlorfenapyr blood concentrations in patients 1, 2, and 3 decreased. Patients one and three were discharged on the 30th and 14th days of admission, respectively, while patient 2, whose condition worsened, died on the 11th day of treatment after unsuccessful resuscitation.

**Conclusion:**

Patients with occupational chlorfenapyr poisoning may have no obvious clinical symptoms or may present with excessive sweating and fatigue in the early stages, unlike patients who have ingested chlorfenapyr orally. Therefore, early detection and imaging examinations are crucial for an accurate diagnosis.

## 1 Introduction

Chlorfenapyr is a novel pyrrole-based insecticide precursor with strong contact and stomach toxicity, effectively killing insects and mites on various crops by disrupting mitochondrial oxidative phosphorylation and reducing adenosine triphosphate (ATP) production (Zhao et al., 2017). Structurally, chlorfenapyr undergoes metabolic activation to form tralopyril, which are the primary bioactive intermediates responsible for its uncoupling effects on mitochondrial membranes (Chung et al., 2022). With the widespread use of chlorfenapyr, cases of chlorfenapyr poisoning have increased in recent years, with most occurring through oral ingestion, although cases of occupational exposure, dermal contact, and intraperitoneal injection have also been reported (Lee et al., 2013; Han et al., 2019; An et al., 2024). Common initial symptoms include sweating, nausea, vomiting, and altered consciousness, while subsequent fever may indicate a poor prognosis. However, no consensus exists regarding the clinical course of chlorfenapyr poisoning, although clinical manifestations and auxiliary examinations have been described in most reported cases (Comstock et al., 2024).

In July 2024, we admitted four cases of occupational chlorfenapyr poisoning from the same chlorfenapyr production plant; three were workshop operators and the other was a product inspector. All four patients underwent multiple blood toxicant tests; three were hospitalized, while the remaining patient was monitored through outpatient observation. After comprehensive treatment of the three hospitalized patients, two were discharged, and one died. The outpatient was monitored and tested positive for the toxicant but exhibited no clinical manifestations. Herein, we report four cases of chlorfenapyr poisoning.

## 2 Materials and methods

The clinical data of four patients with occupational chlorfenapyr poisoning were collected from electronic medical records and retrospectively analyzed. This study was approved by the Ethics Committee of the Qilu Hospital of Shandong University (Jinan, Shandong Province) (Ethics No. KYLL-202106(KS)-040). Written informed consent was obtained from all relevant patients and/or their legal representatives, who signed paper-based informed consent forms.

### 2.1 General patient information

Before admission, all four patients worked in the same factory, which produced chlorfenapyr using p-chlorophenylglycine as the main raw material. Three of them worked in the centrifugal drying workshop, where the ambient temperature was 38°C or higher. The ventilation facilities consisted of industrial ventilation fans, and the workers were equipped with masks, face shields, rubber gloves, and non-disposable protective clothing. The remaining patients worked in product inspection departments. Patient 1, a 23-year-old male, had been working in the workshop for 3 weeks. On 2024-07-07, he developed a headache and sweating and was diagnosed with heat stroke at a local clinic. He was treated for 3 days with no improvement in symptoms and was subsequently transferred to the local hospital for routine blood tests, liver and kidney function tests, magnetic resonance imaging (MRI), and other examinations. On 2024-07-13, he came to our hospital and was admitted to our department. Patient 2, a 32-year-old male, had been working in the workshop for 12 weeks. On 2024-07-17, he presented with symptoms of heavy sweating and heat intolerance and came to our hospital, where he was admitted on 2024-07-22. Patient 3, a 59-year-old male, had worked in the workshop for 16 weeks without experiencing excessive sweating, fatigue, or other discomforts. He visited our hospital on 2024-07-23 for a chlorfenapyr toxicant test, which showed blood chlorfenapyr levels of 0.156 μg/mL and blood tralopyril levels of 0.801 μg/mL. He returned to our hospital on 2024-07-27 and was admitted to our department. Patient 4, a product inspector at the company, was informed of poisoning cases at the company and visited the hospital for toxicant testing. Toxicant testing results showed blood chlorfenapyr levels of <0.005 μg/mL and blood tralopyril levels of 0.025 μg/mL. The patient was followed up in the outpatient clinic for observation, as she did not have any symptoms. Patients 1, 2, and four were previously in good health, and patient three had a history of hypertension for more than 10 years.

### 2.2 Clinical characteristics and treatment

**FIGURE 1:**
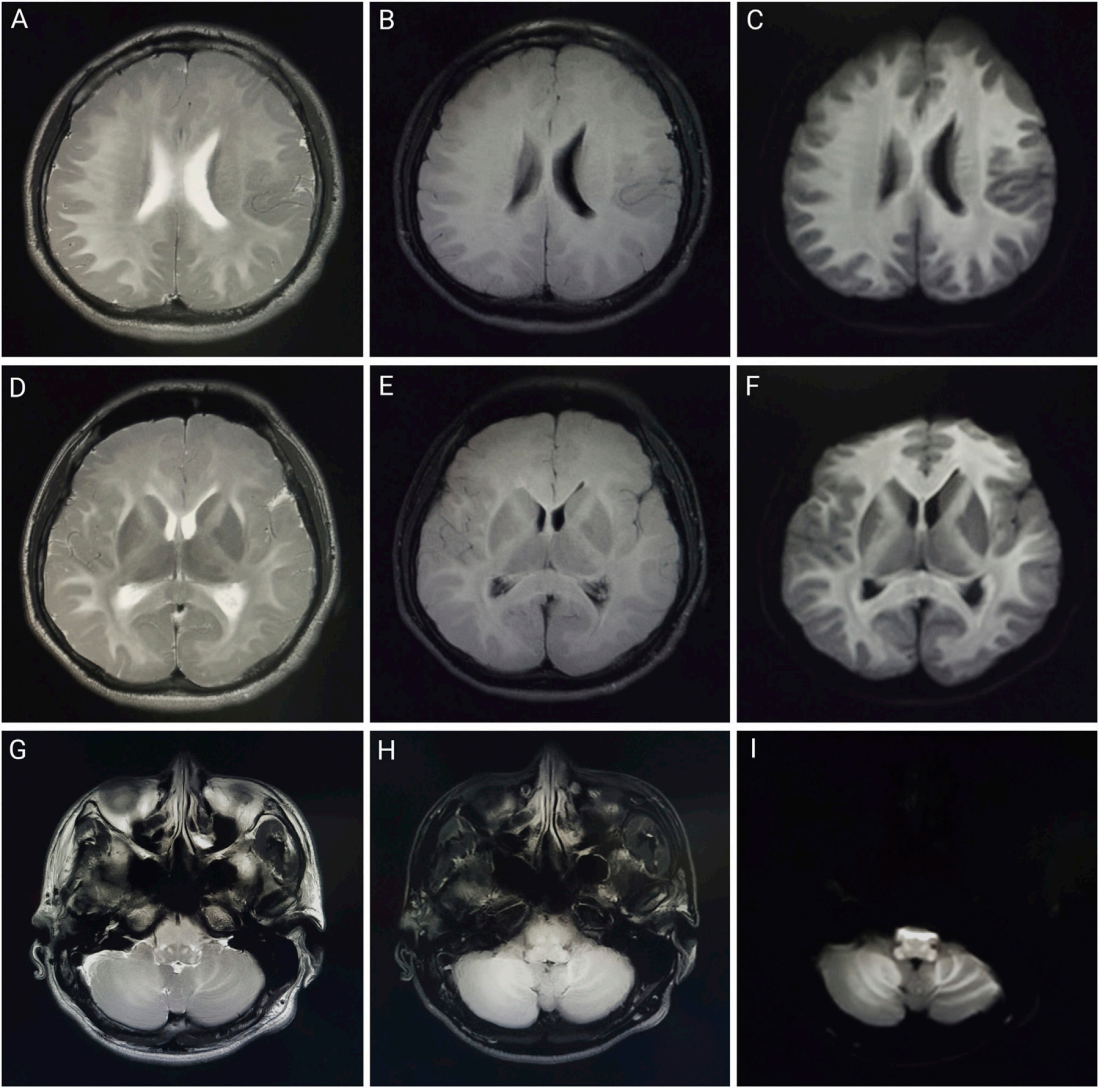
Brain MRI of patient one on day 1 of admission. Brain magnetic resonance imaging of a 23-year-old man after working in a workshop for 3 weeks. Abnormalities included diffuse symmetrical abnormal signals in the bilateral cerebral hemispheres, bilateral cerebellar hemispheres, and brainstem. **(A,D,G)** are T2-weighted images; **(B,E,H)** are T2 fluid-attenuated inversion recovery (FLAIR) images; **(C,F,I)** are diffusion-weighted images.

**FIGURE 2:**
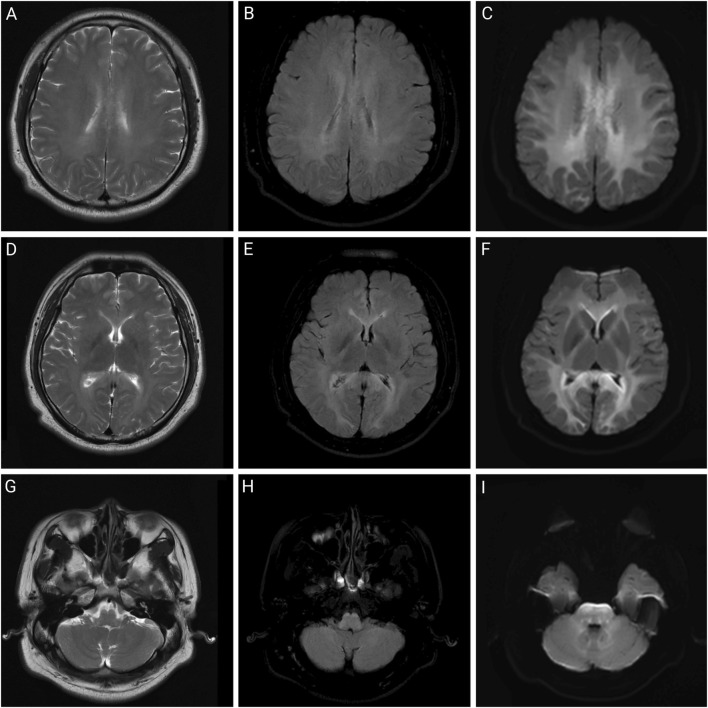
Brain MRI of patient two on day 2 of admission. Brain magnetic resonance imaging of a 32-year-old man after working in a workshop for 3 months. Abnormalities include symmetrical abnormal signals in the white matter areas of the bilateral cerebral hemispheres, corpus callosum, internal and external capsule areas, brainstem, and dentate nucleus. **(A,D,G)** are T2-weighted images; **(B,E,H)** are T2 fluid-attenuated inversion recovery (FLAIR) images; **(C,F,I)** are diffusion-weighted images.

None of the patients were febrile at the time of admission, and two (patients one and two) presented with sweating. After admission, patient 2’s condition gradually worsened, and he developed fever, bilateral lower-extremity weakness, coma, and respiratory and circulatory failures. Table 1 presents the primary clinical data of the four patients. Laboratory tests revealed varying degrees of elevated white blood cell counts, neutrophil ratios, and inflammatory factor levels. Transaminase levels were mildly elevated in two patients (patients one and three), urea nitrogen was mildly elevated in all patients, and creatine kinase levels were mildly elevated in one patient (patient 2). The initial concentrations of chlorfenapyr on admission were 0.084, 0.392, and 0.095 μg/mL (reference range <0.005 μg/mL), and the initial concentrations of tralopyril were 3.230, 1.530, and 0.265 μg/mL (reference range <0.001 μg/mL), respectively. The results of major laboratory tests are presented in Table 2. The MRI results of patients 1, 2, and three are shown in Table 1. Patients one and two mainly showed bilateral diffuse symmetrical abnormal signal changes (Figures 1, 2).

**TABLE 1:** The main clinical data and MRI results of the four patients.

Items	Sex	Age	Length of service	Underlying disease	Initial symptoms	Vital signs	Brain imaging findings	Treatment time and outcome
Case 1	Male	23	3 weeks	—	Hyperhidrosis, headache, emaciation, low spirit	T: 36.5°C PR: 70 b/m RR: 19 b/m BP: 134/62 mmHg	MRI: Diffuse symmetrical abnormal signals in bilateral cerebellar hemispheres, bilateral cerebral hemispheres, and brainstem.	30 days, cured.
Case 2	Male	32	3 months	—	Hyperhidrosis, fear of heat	T: 36.5°C PR: 89 b/m RR: 20 b/m BP: 114/64 mmHg	MRI: Symmetrical abnormal signals in white matter areas of bilateral cerebral hemispheres, corpus callosum, internal and external capsule areas, brainstem, and dentate nucleus	11 days, dead.
Case 3	Male	59	4 months	Hypertension	No obvious discomfort	T: 35.9°C PR: 87 b/m RR: 17 b/m BP: 166/117 mmHg	MRI: A little ischemic lesion of white matter in both cerebral hemispheres	14 days, cured.
Case 4	Female	20	—	—	No obvious discomfort	—	CT: No apparent abnormality.	—

PR, pulse rate; RR, respiratory rate; BP, blood pressure; bpm, beats/min.

**TABLE 2:** Results of major laboratory tests at different times after admission.

Items	Time	Case 1	Case 2	Case 3	Case 4
WBC (3.5–9.5 × 109/L)	Day 1	6.55	8.60	3.61	**10.64**
	Day 3	9.30	8.20	8.64	—
	Day 7	9.31	**13.52**	9.04	—
	Day 14	**15.54**	—	7.12	—
NEU% (40.0%–75.0%)	Day 1	79.10	68.20	54.40	67.60
	Day 3	73.60	72.60	**79.00**	**—**
	Day 7	78.80	73.30	74.80	—
	Day 14	**90.90**	—	70.30	—
HGB (115.0–150.0 g/L)	Day 1	145.0	143.0	131.0	137.0
ALT (7.0–40.0 U/L)	Day 1	17.0	31.0	45.0	19.0
	Day 3	9.0	20.0	25.0	—
	Day 7	38.0	19.0	**83.0**	**—**
	Day 14	**86.0**	—	32.0	—
AST (13.0–35.0 U/L)	Day 1	37.0	39.0	45.0	18.0
	Day 3	17.0	17.0	19.0	—
	Day 7	30.0	16.0	35.0	—
	Day 14	31.0	—	15.0	—
TBIL (5.0–21.0 μmol/L)	Day 1	19.0	11.0	9.0	9.8
BUN (2.30–7.80 mmol/L)	Day 1	**7.90**	6.40	7.40	4.09
	Day 3	9.43	6.46	5.55	—
	Day 7	7.97	**12.37**	**8.15**	**—**
	Day 14	5.28	—	6.24	—
Cr (53.0–97.0 μmol/L)	Day 1	60.0	65.0	61.0	55
CK (38–174 U/L)	Day 1	63	—	—	67
	Day 3	146	**200**	80	—
	Day 7	73	49	93	—
	Day 14	33	—	56	—
LDH (120–230 U/L)	Day 1	186	—	—	181
	Day 3	186	200	195	—
	Day 7	**293**	**257**	169	—
	Day 14	193	—	183	—
PT (8.8–13.8 s)	Day 1	11.3	13.0	13.1	11.3
APTT (26.0–42.0)	Day 1	25.6	28.5	37.8	29.1

The abnormal test results are indicated in bold.

Day 1, Poisoning Day; WBC, white blood cell; NEU, neutrophils; HGB, hemoglobin; ALT, alanine aminotransferase; AST, aspartate aminotransferase; TBIL, total bilirubin; BUN, blood urea nitrogen; Cr, serum creatinine; CK, creatine kinase; LDH, lactate dehydrogenase; PT, prothrombin time; APTT, activated partial thromboplastin time.

Currently, there is no specific antidote for chlorfenapyr poisoning, and treatment is based on comprehensive therapy. Commonly used therapeutic drugs include betamethasone (2 mL, qd, intravenous drip), furosemide (20 mg bid, intravenous injection), magnesium isoglycyrrhizinate (200 mg qd, intravenous drip), and polyene phosphatidylcholine (20 mL, qd, intravenous drip). At the same time, HA330 hemoperfusion plus continuous renal replacement therapy (CRRT) was given, which was performed once a day for five consecutive days. Each hemoperfusion lasted for 2 h, and CRRT lasted for 4 h. On 2024-7-30, after five rounds of hemoperfusion plus CRRT, Patient two became comatose, with hyperthermia and dyspnea; blood oxygen saturation dropped to 89%–92%, and the respiratory rate was 32 beats/min. The patient underwent endotracheal intubation and ventilator-assisted respiration, and physical hypothermia and CRRT were administered simultaneously. At 20:06 on 2024-8-2, transient ventricular fibrillation occurred, followed by heart rate monitoring. Cardiopulmonary resuscitation and other resuscitation measures were immediately administered. The patient died of cardiac arrest.

## 3 Results

**FIGURE 3:**
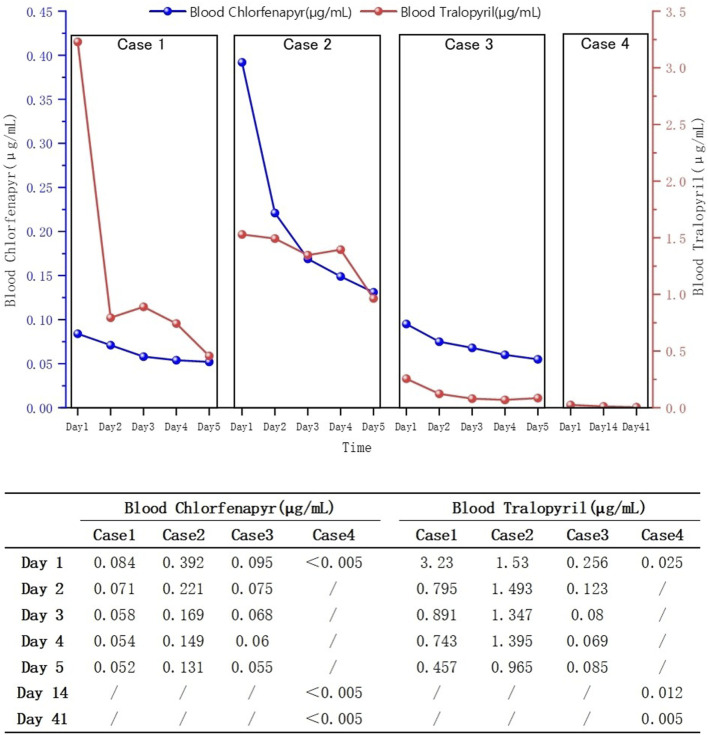
Daily changes in blood chlorfenapyr and tralopyril concentrations of the four patients.

Blood concentrations decreased in all patients (Figure 3). Patients one and three were discharged from the hospital after the 30th and 14th day of treatment, respectively. Patient two died on the 11th day of treatment. At the 1-month follow-up after discharge, blood tests of patients one showed no significant abnormalities and the cranial MRI revealed no evidence of abnormal signals. During the 2-week post-discharge evaluation, blood parameters of patients three remained within normal limits. At the 3-month telephone follow-up, the patient four reported no new symptoms or complications.

## 4 Discussion

Chlorfenapyr is a pro-insecticide that removes n-ethoxymethyl via the monooxygenase system of insect microsomes, transforming it into the toxic metabolite tralopyril (Chung et al., 2022). Tralopyril can uncouple the oxidative phosphorylation of mitochondria, resulting in altered mitochondrial membrane potential and limited ATP synthesis, which affects the organism’s energy metabolism and leads to cellular dysfunction and death (Xia et al., 2018). As the insecticidal mechanism of chlorfenapyr differs from that of traditional insecticides, it is a good alternative to pyrethroids, organophosphates, and other insecticides (Raghavendra et al., 2011; Zhao et al., 2017). In humans, chlorfenapyr can be ingested via oral administration, dermal absorption, or inhalation.

In previous case reports, except for a few cases of occupational exposure, dermal absorption, and intraperitoneal injection, most chlorfenapyr poisoning cases resulted from oral ingestion (Lee et al., 2013; Han et al., 2019; An et al., 2024). As chlorfenapyr mainly affects the energy metabolism of the body, organs with high energy demands, such as the brain, heart, and skeletal muscles, are greatly affected. Common clinical manifestations upon admission include weakness, dizziness, headache, sweating, nausea, and vomiting. As the disease progresses after admission, altered consciousness, fever, rhabdomyolysis, tachycardia, and respiratory and circulatory failure may occur (Comstock et al., 2024). Fever may be a characteristic clinical manifestation of chlorfenapyr poisoning, and its appearance is often accompanied by disease deterioration, suggesting a potential link to prognosis. The appearance of fever may be related to the disruption of ATP production. Tralopyril, the active metabolite of chlorfenapyr, can uncouple the oxidative phosphorylation of mitochondria, leading to reduced ATP synthesis and conversion of electrochemical potential energy into heat energy, resulting in increased body temperature. If chlorfenapyr damages the brain parenchyma, it can affect thermoregulation, causing central fever and potentially leading to respiratory and circulatory failure (Chien et al., 2022). Brain damage caused by chlorfenapyr may appear as bilateral diffuse symmetrical abnormal signal changes on MRI, mainly involving the white matter, which may be related to myelin swelling, vacuolization, and demyelination (Baek et al., 2016). Additionally, animal studies in mice have shown that chlorfenapyr can reduce the oxidation resistance of the liver, induce oxidative stress, and cause hepatocyte damage, ultimately leading to liver damage. This study also showed that chlorfenapyr disrupted the intestinal immune balance and caused intestinal inflammation in mice (Xiong et al., 2024). Moreover, the patients in this study showed varying degrees of altered consciousness and sweating during hospitalization, some of whom had mild elevations in transaminases, urea nitrogen, and creatine kinase levels. Patient two was exposed to a high concentration of chlorfenapyr nitrile and developed fever, bilateral lower-extremity weakness, tachycardia, and respiratory and circulatory failure. The MRIs of patients one and two showed symmetrically high signals on T2WI, T2FLAIR, and diffused-weighted imaging in the white matter areas of the bilateral cerebral hemispheres, cerebellum, and brainstem, consistent with toxic leukoencephalopathy. Since the patients in this study were poisoned due to occupational exposure, they did not show gastrointestinal irritation symptoms such as nausea and vomiting.

Limited information is available on the toxicity of chlorfenapyr in humans, and no toxicokinetic studies have been conducted on its toxicity in humans. Based on a small number of published case reports and studies, chlorfenapyr is moderately toxic to rats and mice by oral and inhalation routes but less toxic to rabbits via dermal contact. The oral LD50 values are 441 mg/kg in male rats and 45 mg/kg in male mice, the dermal LD50s is >2,000 mg/kg in rabbits, and the inhalation LD50 is 0.83 mg/L in male rats (Food and Agriculture Organization and Wold Health Organization, 2019). Although chlorfenapyr is classified as a moderately toxic insecticide by the World Health Organization, the fatality rate of chlorfenapyr poisoning is as high as 76% (Comstock et al., 2024). Increasing evidence suggests that chlorfenapyr damages the brain, heart, skeletal muscle, and other organs and that there is a latency period for the onset of clinical symptoms, possibly related to its hepatic metabolism into the active substance tralopyril (Tharaknath et al., 2013). Therefore, even if patients with chlorfenapyr poisoning have mild early symptoms and normal laboratory and imaging results, the possibility of a poor prognosis cannot be excluded.

The clinical diagnosis of chlorfenapyr poisoning is often based on history-taking, clinical manifestations, and circumstantial evidence. Toxicant testing for chlorfenapyr is rarely performed. Liquid chromatography-tandem mass spectrometry has high sensitivity and resolution and can be used to determine the concentrations of chlorfenapyr and its metabolite, tralopyril (Viette et al., 2011). Patients 1, 2, and three underwent daily hemoperfusion plus CRRT for 5 days after admission. Meanwhile, plasma chlorfenapyr and tralopyril concentrations were measured, showing a decreasing trend in the plasma concentrations of chlorfenapyr in all three patients. Although tralopyril concentrations generally showed a downward trend, a rebound was observed during the period, likely due to the decrease of toxicant in the blood and its re-entry from the tissues. On the first day of admission, patient one had the highest concentration of tralopyril among the three patients; however, tralopyril levels, a metabolite of chlorfenapyr, were affected by the concentration of chlorfenapyr. Since patient one was admitted to the hospital with a low concentration of chlorfenapyr, his tralopyril concentration decreased significantly and fluctuated at a lower level after the first day of blood purification treatment. The blood concentrations of chlorfenapyr and tralopyril at admission did not show a clear correlation with the duration of occupational exposure. This may have been affected by several factors, such as protective measures, metabolism rate of the toxins, recall bias, and sample size. However, a correlation was observed between the severity of poisoning in the patients and the concentrations of chlorfenapyr and tralopyril in the blood. Patient two had the most severe poisoning and the highest chlorfenapyr concentration levels. Patient three did not show obvious symptoms of poisoning during hospitalization, and the concentrations of chlorfenapyr and tralopyril were the lowest among the three patients. Furthermore, this study, through comparative analysis of clinical characteristics and imaging findings in four cases of chlorfenapyr poisoning, demonstrates the potential clinical value of MRI in assessing the severity of intoxication and prognostic evaluation. Patients one and two, who presented with prominent toxic symptoms upon admission—including hyperhidrosis, dreading heat, and altered mental status—exhibited extensive white matter abnormalities on MRI, predominantly involving bilateral cerebral hemispheric white matter, corpus callosum, brainstem, and cerebellum. In contrast, Patient 3, with mild clinical symptoms, showed no significant white matter signal abnormalities. The selective vulnerability of white matter observed in these cases may be attributed to the neurotoxic mechanisms of chlorfenapyr, which is hypothesized to lead to energy metabolism dysfunction. White matter, characterized by high energy demands due to its myelinated structures, appears particularly susceptible to such metabolic disturbances, which may explain the preferential white matter injury. From a prognostic perspective, the extent of white matter abnormalities correlated with clinical severity. Patient one demonstrated a parallel reduction in white matter lesion volume on follow-up MRI and concurrent clinical improvement, suggesting that early MRI evaluation may not only aid in diagnosis but also serve as a prognostic marker. In contrast, patient three achieved recovery in the absence of significant MRI abnormalities, suggesting that mild poisoning may not be accompanied by structural brain damage.

No specific antidote exists for chlorfenapyr poisoning, and treatment is based on symptomatic supportive therapy. Patients who take chlorfenapyr orally, with a clear history of ingestion and prominent gastrointestinal symptoms, are often hospitalized promptly. However, early poisoning symptoms due to occupational exposure are not obvious, potentially delaying timely intervention. When patients experience sweating, weakness, and dizziness, chlorfenapyr poisoning may be mistaken for other diseases, such as heatstroke. Therefore, obtaining occupational histories, conducting toxicant tests, and improving auxiliary examinations are particularly important for the early diagnosis of chlorfenapyr poisoning.

This research, however, is subject to several limitations that should be acknowledged. First, as a descriptive study, it is constrained by the rarity of chlorfenapyr exposure incidents, which resulted in a relatively small sample size. The limited number of cases precluded comparative analyses with similar incidents and robust statistical validation, potentially affecting the generalizability of the findings. Second, all samples in this study were derived from a single chlorfenapyr exposure event. Consequently, environmental factors specific to the patients’ workplace–such as ambient temperature and ventilation conditions–in addition to individual differences in the use of personal protective equipment may have influenced the outcomes. These context-specific variables limit the extrapolation of our results to other settings. Third, the observation period for this study was confined to less than 1 year, which restricted us to evaluate long-term health effects associated with chlorfenapyr exposure. Moving forward, we intend to accumulate more cases in future clinical work to enhance the reliability of our conclusions, collect patients from diverse exposure occasions to assess the stability and broader applicability of our findings and implement sustained follow-up protocols to monitor the health trajectories of the patients over an extended timeframe.

## 5 Conclusion

With the accumulation of toxins, patients with occupational chlorfenapyr poisoning may gradually develop symptoms such as sweating, dizziness, and weakness. Since clinical manifestations have a latent period, patients must be closely monitored. Even if early symptoms are mild and laboratory test results are normal, the possibility of a poor prognosis cannot be excluded. As the disease progresses, patients may develop altered consciousness, fever, rhabdomyolysis, tachycardia, and respiratory and circulatory failure. Toxicant detection of chlorfenapyr and its metabolite tralopyril can help confirm the diagnosis in patients with unknown insecticide exposure and assess the severity of poisoning. Early MRI examination can help clarify the damage to the central nervous system caused by chlorfenapyr, determine the prognosis, and guide treatment.

## Data Availability

The original contributions presented in the study are included in the article/supplementary material, further inquiries can be directed to the corresponding author.

## Abbreviations

ATP, Adenosine Triphosphate; CRRT, Continuous Renal Replacement Therapy; HA330, Hemoperfusion Adsorber 330; MRI, Magnetic Resonance Imaging; qd, Quaque Die; bid, Bis in Die.
